# Insurance Instability for Patients With Opioid Use Disorder in the Year After Diagnosis

**DOI:** 10.1001/jamahealthforum.2024.2014

**Published:** 2024-07-26

**Authors:** Paul J. Christine, Anna L. Goldman, Jake R. Morgan, Shapei Yan, Avik Chatterjee, Amy L. Bettano, Ingrid A. Binswanger, Marc R. LaRochelle

**Affiliations:** 1 Division of General Internal Medicine, Department of Medicine, University of Colorado School of Medicine, Aurora; 2 Department of General Internal Medicine, Denver Health and Hospital Authority, Denver, Colorado; 3 Section of General Internal Medicine, Department of Medicine, Boston Medical Center, Boston, Massachusetts; 4 Section of General Internal Medicine, Department of Medicine, Chobanian and Avedisian School of Medicine, Boston University, Boston, Massachusetts; 5 Department of Health Law, Policy, and Management, Boston University School of Public Health, Boston, Massachusetts; 6 Office of Population Health, Massachusetts Department of Public Health, Boston,; 7 Institute for Health Research, Kaiser Permanente Colorado, Aurora; 8 Colorado Permanente Medical Group, Denver; 9 Department of Health Systems Science, Kaiser Permanente Bernard J. Tyson School of Medicine, Pasadena, California

## Abstract

**Importance:**

Transitions in insurance coverage may be associated with worse health care outcomes. Little is known about insurance stability for individuals with opioid use disorder (OUD).

**Objective:**

To examine insurance transitions among adults with newly diagnosed OUD in the 12 months after diagnosis.

**Design, Setting, and Participants:**

Longitudinal cohort study using data from the Massachusetts Public Health Data Warehouse. The cohort includes adults aged 18 to 63 years diagnosed with incident OUD between July 1, 2014, and December 31, 2014, who were enrolled in commercial insurance or Medicaid at diagnosis; individuals diagnosed after 2014 were excluded from the main analyses due to changes in the reporting of insurance claims. Data were analyzed from November 10, 2022, to May 6, 2024.

**Exposure:**

Insurance type at time of diagnosis (commercial and Medicaid).

**Main Outcomes and Measures:**

The primary outcome was the cumulative incidence of insurance transitions in the 12 months after diagnosis. Logistic regression models were used to generate estimated probabilities of insurance transitions by insurance type and diagnosis for several characteristics including age, race and ethnicity, and whether an individual started medication for OUD (MOUD) within 30 days after diagnosis.

**Results:**

There were 20 768 individuals with newly diagnosed OUD between July 1, 2014, and December 31, 2014. Most individuals with newly diagnosed OUD were covered by Medicaid (75.4%). Those with newly diagnosed OUD were primarily male (67% in commercial insurance, 61.8% in Medicaid). In the 12 months following OUD diagnosis, 30.4% of individuals experienced an insurance transition, with adjusted models demonstrating higher transition rates among those starting with Medicaid (31.3%; 95% CI, 30.5%-32.0%) compared with commercial insurance (27.9%; 95% CI, 26.6%-29.1%). The probability of insurance transitions was generally higher for younger individuals than older individuals irrespective of insurance type, although there were notable differences by race and ethnicity.

**Conclusions and Relevance:**

This study found that nearly 1 in 3 individuals experience insurance transitions in the 12 months after OUD diagnosis. Insurance transitions may represent an important yet underrecognized factor in OUD treatment outcomes.

## Key Points

**Question:**

What is the incidence of insurance transitions among individuals with opioid use disorder covered by commercial insurance or Medicaid in the first 12 months after their diagnosis?

**Findings:**

In this cohort study of 20 768 individuals in Massachusetts with newly diagnosed opioid use disorder in 2014, 30.4% of individuals experienced an insurance transition in the 12 months after diagnosis, with slightly higher transition rates in Medicaid compared with commercial insurance.

**Meaning:**

Results of this study suggest that insurance transitions in individuals with opioid use disorder are common and may represent an important yet underrecognized factor in treatment outcomes.

## Teaser

This cohort study estimates the cumulative incidence of insurance transitions among patients with newly diagnosed opioid use disorder covered by Medicaid or commercial insurance in the first year after their diagnosis.

## Introduction

In the US, health insurance is an important determinant of health, enabling access to diagnostic and therapeutic services that can improve outcomes.^1,2,3,4,5^ Researchers and policy makers have long focused on increasing enrollment in health insurance as a means to improve health. Enrolling in insurance may have more limited effects, however, if individuals are unable to maintain their insurance. Research on insurance churn, defined as the movement of individuals between or out of insurance plans, has highlighted the importance of maintaining continuous insurance coverage and minimizing insurance transitions.^6,7^ Gaps in insurance coverage have been linked to delayed medical care, decreased medication adherence, and increased financial risk.^8,9,10^ Even without a gap in coverage, switching insurance plans may result in treatment disruptions due to differences in benefits or clinician practices.^7,10,11^ In contrast, insurance transitions could potentially lead to improvements in clinician network breadth and to more generous benefits.^12,13,14^

Opioid use disorder (OUD) is a chronic condition associated with significant morbidity and mortality, with more than 80 000 opioid-related overdose deaths in the US in 2021 alone.^15^ There are multiple effective medications for OUD (MOUD) associated with decreased morbidity and mortality, although they remain underused.^16,17^ Insurance benefits are crucial for accessing life-saving MOUD, as the cost of these medications can be prohibitive for patients with OUD with disproportionately low income.^18,19,20^ Furthermore, treatment retention in MOUD is associated with decreased mortality,^16^ but insurance instability may limit retention if patients cannot afford services. Despite the potential importance of insurance stability to treatment access and retention for OUD, there has been limited research investigating insurance stability in this patient population.

Using data from the Massachusetts Public Health Data Warehouse, we sought to estimate the cumulative incidence of insurance transitions among patients with newly diagnosed OUD covered by Medicaid or commercial insurance in the first year after their diagnosis. We also estimated the probability of insurance transitions across various sociodemographic groups, given known inequities in access to MOUD.^21,22^

## Methods

### Study Design and Data Source

We performed a retrospective cohort study using data from the Massachusetts Public Health Data Warehouse. In the Public Health Data Warehouse, records from the All-Payer Claims Database (APCD) are linked longitudinally to individual-level administrative records from more than 30 sources using a probabilistic linkage which has been described previously.^23^ For this study, we used data from the APCD, Acute Care Hospital Case Mix, the Prescription Monitoring Program, the Bureau of Substance Addiction Services, the Registry of Vital Records and Statistics, the state Department of Correction (state prisons), and county Houses of Corrections (county jails) (eTable 1 in Supplement 1). The Public Health Data Warehouse suppresses the output for all counts between 1 and 10 to protect privacy. This study was determined to be not human participant research by the Boston University Medical Campus Institution Review Board. The study followed the Strengthening the Reporting of Observational Studies in Epidemiology (STROBE) reporting guideline.

### Reporting of Race and Ethnicity

The Department of Public Health sought to identify race and ethnicity for as many individuals as possible included in the Massachusetts Public Health Data Warehouse. For each data set with race and ethnicity, individuals were assigned 1 of 5 mutually exclusive race categories of Asian/Pacific Islander or Native Hawaiian non-Hispanic, Black non-Hispanic, Hispanic, White non-Hispanic, non-Hispanic American Indian, Alaska Native, or other. The Massachusetts Public Health Data Warehouse then extracted a single race and ethnicity for each individual using a hierarchy of which data sets most reliably used self-reported race and ethnicity. In the case of more than one race being reported, we used the following approach: take the most frequently reported race and ethnicity for that individual, and if there was a tie for the most frequently reported race and ethnicity for that individual, and one of those tied values was Hispanic, then the individual was identified as Hispanic. Otherwise, individuals were assigned as the least common race in Massachusetts that they had listed as one of their tied race categories with the following configuration: the least common were American Indian/Alaska Native/Other, non-Hispanic; second least common, Asian, Pacific Islander, Native Hawaiian, non-Hispanic; third least common, Black, non-Hispanic, and most common, White, non-Hispanic.

### Cohort Selection

We defined a cohort of Massachusetts residents with incident OUD using *International Classification of Diseases, Ninth Revision (ICD-9)* and *International Statistical Classification of Diseases and Related Health Problems, Tenth Revision (ICD-10)* diagnosis codes for insurance claims submitted to the Massachusetts APCD and Acute Care Hospital Case Mix files (eTables 1 and 2 in Supplement 1). We included adults aged 18 to 63 years to exclude children and individuals who would become eligible for Medicare during follow-up. We defined an incident diagnosis as those without a diagnosis or OUD-related claim in the previous 180 days. Because most individuals with OUD in Massachusetts are insured by either Medicaid or commercial insurers (80.1% during the study timeframe), we focused on these 2 types of insurance at the time of diagnosis. The primary cohort included individuals diagnosed between July 1, 2014, and December 31, 2014, with follow-up through December 31, 2015. At the start of 2016, APCD reporting requirements were removed for self-funded employer plans governed by the Employee Retirement Income Security Act. These changes resulted in an abrupt 27% decrease in commercial claims submitted to the APCD in early 2016, which stabilized after the start of the year.^24^ To minimize this change in reporting requirements in our estimates, we excluded individuals with incident diagnoses after 2014 and restricted our follow-up period to 2015.

### Exposures and Outcomes

The exposure of interest was having commercial or Medicaid insurance at the time of diagnosis. Our main outcome of interest was the incidence of insurance transitions in the 12 months after diagnosis. Incident insurance transition was defined as any transition away from baseline insurance type at time of diagnosis (either Medicaid or commercial) into a different insurance type or having no reported insurance during follow-up. We used the Massachusetts APCD to classify mutually exclusive insurance types each month. Our secondary outcome was the type of insurance transition experienced. While individuals were restricted to having commercial or Medicaid insurance at the time of diagnosis, they could transition to any insurance type, including commercial (from Medicaid), Medicaid (from commercial), Medicare Advantage, Emergency Medicaid/other (including Veterans Affairs and worker’s compensation), or uninsured/missing. The uninsured/missing category includes individuals who became uninsured, switched to an insurer that does not report to the APCD (eg, fee-for-service Medicare), moved out of state, died, or became incarcerated. We lacked data on the components of the missing category except for death and incarceration.

To characterize the study cohort, we abstracted the following information at the time of diagnosis: age category (aged 18-25, 26-34, 35-44, and 45-63 years); sex; race and ethnicity (with categories as given in the Massachusetts Public Health Data Warehouse as Asian/Pacific Islander non-Hispanic, Black non-Hispanic, Hispanic, White non-Hispanic, other non-Hispanic, missing); pregnancy status (yes/no); number of hospitalizations and emergency department visits in the 180 days prior to diagnosis (0, 1, 2, ≥3); incarceration in the 180 days prior to diagnosis (yes/no); whether an individual experienced an opioid overdose in the 180 days prior to diagnosis (yes/no); and whether they started MOUD (buprenorphine, methadone, naltrexone) in the 30 days after diagnosis (yes/no). We included race and ethnicity as a marker of experiences of discrimination and structural racism that individuals face.

### Statistical Analysis

We calculated descriptive statistics at the time of diagnosis stratified by commercial vs Medicaid insurance. We then calculated the unadjusted cumulative incidence of experiencing a first insurance transition during the 12 months after diagnosis by insurance type using the Kaplan-Meier estimator. We then tabulated the frequency and percentage of insurance transition types (transitions from commercial or Medicaid at baseline to commercial, Medicaid, Medicare Advantage, other, or uninsured/missing).

To characterize patient characteristics associated with insurance transitions in the 12 months after diagnosis, we used logistic regression models to generate adjusted estimated probabilities of insurance transitions using a priori selected estimators based on the literature: age category, race and ethnicity, pregnancy status, and whether individuals started MOUD in the 30 days after diagnosis.^25,26^ All logistic regression models were run separately by insurance type, except for models investigating the probability of insurance transitions by baseline insurance type, which pooled all patients together.

We also performed several sensitivity analyses. First, because individuals may lose insurance temporarily due to lapses in paperwork, we performed additional analyses where we allowed individuals to transition from their baseline insurance to the uninsured/missing category for a month, and then return to their baseline insurance without counting it as an insurance transition. Using this 2-month definition, we recalculated the unadjusted cumulative incidence of insurance transitions and the types of insurance transitions that occurred. Second, to clarify whether individuals who transitioned to the uninsured/missing category later regain insurance, we calculated the frequency with which these individuals transitioned back to another insurance category by the end of 12-month follow-up. Third, to assess how insurance transitions may have changed over time, we expanded the cohort to use more recent data, including individuals diagnosed with OUD from July 2014 to March 2019 (with follow-up through March 2020). In this expanded cohort, we excluded individuals diagnosed with OUD in 2015 whose follow-up would have spanned the time when APCD reporting requirements changed. Fourth, to help contextualize the insurance transition patterns in patients with OUD, we compared insurance transition incidence and estimated probabilities to a propensity score–matched cohort of individuals with an incident diagnosis of type 2 diabetes (T2D), another chronic condition requiring routine visits and consistent medication.

All analyses were performed using SAS Enterprise Edition software version 3.81 (SAS Institute Inc) and took place from November 10, 2022, to May 6, 2024.

## Results

### Baseline Characteristics

There were 20 768 individuals diagnosed with incident OUD between July 1, 2014, and December 31, 2014 (Table 1; eTable 3 in Supplement 1). Most individuals with newly diagnosed OUD were male (78.4% of those with Medicaid, 87.4% of those with commercial insurance). Just over three-quarters of individuals with OUD (75.4%) had Medicaid insurance at baseline. Of those with Medicaid insurance, 0.4% were Asian/Pacific Islander non-Hispanic, 7.1% were Black non-Hispanic, 12.9% were Hispanic, 78.4% were White non-Hispanic, and 0.9% were other non-Hispanic, and of those with commercial insurance, 0.5% were Asian/Pacific Islander non-Hispanic, 2.6% were Black non-Hispanic, 3.2% were Hispanic, 87.4% were White non-Hispanic, and 2.1% were other non-Hispanic. Individuals with OUD receiving Medicaid also had more hospitalizations and emergency department visits in the 180 days prior to diagnosis compared with those with commercial insurance. Less than one-third of patients with OUD started MOUD within 30 days after diagnosis (28.7% in commercial insurance and 32.7% in Medicaid insurance).

**Table 1.** Patient Characteristics at the Time of OUD Diagnosis, by Commercial vs Medicaid Insurance, 2014-2015

Characteristic	Insurance type, No. (%)	
	Commercial	Medicaid
Sample size	5115 (24.6)	15 653 (75.4)
Age category, y		
18-25	1789 (35.0)	2324 (14.9)
26-35	1207 (23.6)	6048 (38.6)
36-45	869 (17.0)	3392 (21.7)
46-63^a^	1250 (24.4)	3889 (24.9)
Sex		
Female	1690 (33.0)	5980 (38.2)
Male	3425 (67.0)	9673 (61.8)
Race and ethnicity		
Asian/Pacific Islander non-Hispanic	25 (0.5)	61 (0.4)
Black non-Hispanic	134 (2.6)	1111 (7.1)
Hispanic	162 (3.2)	2014 (12.9)
White non-Hispanic	4472 (87.4)	12 274 (78.4)
Other non-Hispanic^b^	106 (2.1)	139 (0.9)
Missing	216 (4.2)	54 (0.3)
Pregnant	DS^c^	36 (0.2)
Hospitalizations in last 180 d, No.		
0	4724 (92.4)	13 682 (87.4)
1	264 (5.2)	1284 (8.2)
2	69 (1.4)	360 (2.3)
≥3	58 (1.1)	327 (2.1)
Emergency department visits in last 180 d, No.		
0	3933 (76.9)	8478 (54.2)
1	766 (15.0)	3431 (21.9)
2	230 (4.5)	1693 (10.8)
≥3	186 (3.6)	2051 (13.1)
Incarcerated in last 180 d^d^	101 (2.0)	1226 (7.8)
Opioid overdose in last 180 d	72 (1.4)	396 (2.5)
Started medication for OUD at time of diagnosis		
None	3649 (71.3)	10 529 (67.3)
Methadone	118 (2.3)	1349 (8.6)
Buprenorphine	1134 (22.2)	3250 (20.8)
Naltrexone	219 (4.3)	564 (3.6)

Abbreviations: DS, data suppressed; OUD, opioid use disorder.

^a^ Cohort limited to adults aged 18 to 63 years. The upper age limit of 63 years was imposed to avoid counting insurance transitions to Medicare at age 65 years.

^b^ Including American Indian and Alaska Native.

^c^ The Public Health Data Warehouse suppresses the output for all counts between 1 and 10 to protect privacy.

^d^ Incarceration data include all state prisons and most county jails (10 of 13), except for Bristol, Barnstable/Nantucket, and Duke counties.

### Incidence of Insurance Transitions

In the first 12 months after diagnosis, 30.4% of patients with OUD experienced a transition in insurance. The unadjusted incidence of insurance transitions was similar for those with Medicaid at baseline (30.7%) compared with those with commercial insurance at baseline (29.4%) (Figure and Table 2). The cumulative incidence of insurance transitions increased most rapidly in the first 6 months after diagnosis (Figure).

**Figure.**
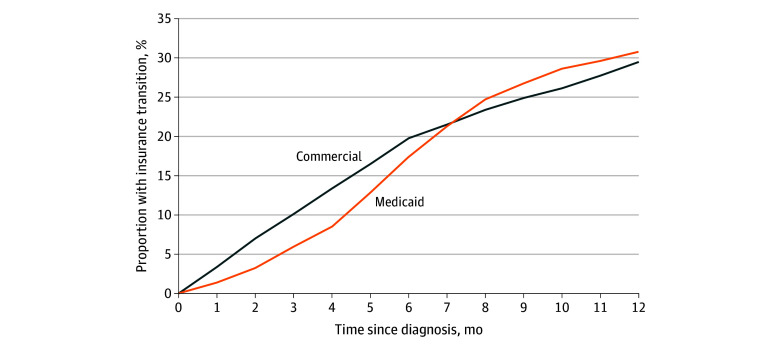
Cumulative Crude Incidence of Insurance Transitions in 12 Months After Opioid Use Disorder Diagnosis, by Commercial and Medicaid Insurance, 2014-2015 Insurance transition was defined as a change from baseline insurance type (either commercial or Medicaid) to either a different type of insurance or missing insurance, which could occur due to moving away from Massachusetts, becoming uninsured, dying, or becoming incarcerated.

**Table 2.** Types of Insurance Transitions Experienced in 12 Months Following Opioid Use Disorder Diagnosis for Individuals With Commercial or Medicaid Insurance, 2014-2015^a^

Insurance transition	Insurance type, No. (%)	
	Commercial at time of diagnosis	Medicaid at time of diagnosis
No transition	3603 (70.6)	10 848 (69.3)
Any transition	1502 (29.4)	4805 (30.7)
Medicaid	486 (9.5)	NA
Commercial	NA	364 (2.3)
Medicare Advantage^b^	11 (0.2)	43 (0.3)
Other	155 (3.0)	575 (3.7)
Missing	850 (16.7)	3823 (24.4)
Died^c^	13 (1.5)	113 (3.0)
Incarcerated^c^	DS^d^	138 (3.6)

Abbreviations: DS, data suppressed; NA, not applicable.

^a^ Insurance transition defined as a change from baseline insurance type (either commercial or Medicaid) to either a different insurance type or missing insurance, which could occur due to moving away from Massachusetts, becoming uninsured, dying, or becoming incarcerated. The other category includes emergency Medicaid, Veterans Affairs, and worker’s compensation.

^b^ Study population limited to individuals younger than 64 years at time of diagnosis. Insurance transitions to Medicare therefore likely indicate enrollment due to the presence of a qualifying disability, kidney failure, or amyotrophic lateral sclerosis.

^c^ Denominator for died and incarcerated is the number of missing rather than the number with a particular insurance. Incarceration data include all state prisons and most county jails (10 of 13), except for Bristol, Barnstable/Nantucket, and Duke counties.

^d^ The Public Health Data Warehouse suppresses the output for all counts between 1 and 10 to protect privacy.

The most common type of insurance transition was to the uninsured/missing category, which occurred in 16.7% of individuals with commercial insurance and 24.4% of individuals with Medicaid (Table 2). Transitions to other forms of insurance were less common, although 9.5% of patients with OUD and commercial insurance transitioned to Medicaid. Among the 24.4% of individuals with OUD who transitioned from Medicaid to the uninsured/missing category, 6.6% died or were incarcerated.

### Estimators of Insurance Transitions

In adjusted logistic regression models, the overall estimated probability of experiencing an insurance transition in the 12 months after OUD diagnosis was 31.3% (95% CI, 30.5%-32.0%) in Medicaid and 27.9% (95% CI, 26.6%-29.1%) in commercial insurance (Table 3, eFigure 1 in Supplement 1). The probability of insurance transitions was generally higher among younger individuals, particularly for those aged 18 to 24 years with Medicaid (39.0%; 95% CI, 37.0%-40.9%) and those aged 26 to 35 years with commercial insurance (37.5%; 95% CI, 34.8%-40.3%). In those with commercial insurance, Black non-Hispanic individuals had the highest probability of insurance transitions at 40.1% (95% CI, 31.8%-48.4%) followed by Hispanic individuals at 33.6% (95% CI, 26.5%-40.8%). For individuals covered by Medicaid, Black non-Hispanic and Hispanic individuals had lower probabilities of insurance transitions than White non-Hispanic individuals (Table 3; eFigure 1 in Supplement 1). The probability of an insurance transition was lower for individuals who started MOUD at the time of diagnosis when covered by Medicaid (27.0% in those starting vs 32.5% in those not starting MOUD) but not when covered by commercial insurance (28.9% in those starting vs 29.7% in those not starting MOUD).

**Table 3.** Estimated Probability of Insurance Transition in the 12 Months After OUD Diagnosis by Insurance Type and Select Patient Characteristics, 2014-2015^a^

Characteristic	Probability of insurance transition (95% CI)	
	Commercial at time of diagnosis	Medicaid at time of diagnosis
Overall	27.9 (26.6-29.1)	31.3 (30.5-32.0)
Age category, y		
18-25	31.1 (28.9-33.2)	39.0 (37.0-40.9)
26-35	37.5 (34.8-40.3)	34.5 (33.3-35.7)
36-45	27.8 (24.8-30.8)	29.5 (28.0-31.0)
46-63	20.6 (18.3-22.9)	21.0 (19.7-22.3)
Race and ethnicity		
Asian/Pacific Islander non-Hispanic	21.7 (5.3-38.1)	34.0 (22.6-45.3)
Black non-Hispanic	40.1 (31.8-48.4)	26.5 (23.9-29.1)
Hispanic	33.6 (26.5-40.8)	25.4 (23.6-27.3)
White non-Hispanic	29.3 (28.0-30.6)	31.8 (31.0-32.6)
Other non-Hispanic^b^	15.7 (8.7-22.7)	32.6 (25.1-40.1)
Missing	30.7 (24.5-36.9)	61.4 (48.7-74.2)
Started medication for OUD at time of diagnosis		
No	29.7 (28.2-31.1)	32.5 (31.6-33.4)
Yes	28.9 (26.6-31.3)	27.0 (25.8-28.2)

Abbreviation: OUD, opioid use disorder.

^a^ Estimated probabilities generated from logistic regression models with insurance transition (yes/no) as the outcome. All models included age categories, race and ethnicity, pregnancy status, and insurance type, and whether an individual started medication for OUD at the time of diagnosis. Apart from generating the overall probabilities for insurance transitions, which included individuals with both commercial and Medicaid insurance in the same model, all regression models were run separately by insurance type (commercial vs Medicaid).

^b^ Including American Indian and Alaska Native.

### Sensitivity Analyses

Sensitivity analyses allowing individuals to enter the missing category for 1 month and then return to their prior insurance without counting it as a transition decreased the rate of insurance transitions more for those with Medicaid (23.5% vs 30.7% in main analyses) than for those with commercial insurance (27.4% vs 29.4% in main analyses) (eTable 4 and eFigure 2 in Supplement 1). In analyses tracking individuals who entered the uninsured/missing category, 74.6% of those with commercial insurance at diagnosis remained uninsured/missing throughout follow-up while 20.5% returned to a commercial plan (eTable 5 in Supplement 1). For those with Medicaid, 47.7% remained uninsured/missing throughout follow-up, and 49.6% reenrolled in Medicaid.

Using the expanded cohort with data from July 2014 through March 2020 resulted in lower 12-month rates of insurance transitions for patients with Medicaid (28.3%) and higher rates for patients with commercial insurance (34.0%) (eTable 6 and eTable 7 in Supplement 1). The estimated probabilities of insurance transitions in this expanded cohort showed patterns similar to the main analyses (eTable 8 in Supplement 1).

In the propensity score–matched cohorts of patients with OUD or T2D, rates of insurance transitions were higher for patients with OUD than T2D, particularly for those with commercial insurance (eTable 9, eTable 10, and eFigure 3 in Supplement 1). For patients covered by Medicaid at baseline, 28.3% of individuals with OUD experienced an insurance transition compared with 25.3% of individuals with T2D (eTable 10 in Supplement 1). For patients with commercial insurance at baseline, 26.8% of patients with OUD experienced an insurance transition compared with 17.5% with T2D. The estimated probabilities of insurance transitions by sociodemographic factors also differed by diagnosis (eTable 11 in Supplement 1).

## Discussion

In this study using longitudinal individual-level data, individuals with OUD had high rates of insurance transitions in the 12 months after their diagnosis. Nearly one-third of individuals experienced an insurance transition, with particularly high rates of transition among young individuals and among individuals with commercial insurance who were Black non-Hispanic and Hispanic. Rates were slightly higher in Medicaid compared with commercial insurance although some Medicaid transitions appeared to be brief interruptions with rapid reenrollment.

To our knowledge, this is the first study to investigate insurance instability in individuals with newly diagnosed OUD. While not directly comparable, Nguyen et al^27^ previously found high rates (24.8%) of insurance disenrollment among patients with OUD actively receiving treatment with buprenorphine in an integrated health insurance and delivery system. The rates of insurance transition among patients with OUD appear to be higher than in the general population in Massachusetts, where an estimated 26% of individuals switched insurers over a 2-year period.^7^ They are also higher than insurance transition rates for individuals with schizophrenia in Massachusetts, of whom 18.6% have at least 1 insurance transition per year.^28^

We found that insurance transitions for patients with OUD varied considerably by age and race and ethnicity. Younger individuals were most likely to experience insurance transitions whether in commercial insurance or Medicaid. Individuals aged 26 to 35 years with commercial insurance were at particularly high risk, perhaps due to less stable employment.^29^ Similar age patterns have been observed in studies of pregnancy and psychosis.^25,30^ In terms of race and ethnicity, Black non-Hispanic individuals with OUD covered by commercial insurance had a notably high rate of insurance transitions at 40.1% followed by Hispanic individuals at 33.6%. This finding is consistent with prior research about racial and ethnic inequities in insurance transitions in the peripartum period.^26^ However, differences were less pronounced for patients with OUD covered by Medicaid. Future research should investigate the clinical impact of insurance transitions among individuals with OUD, particularly among racially minoritized individuals who transition from commercial insurance at a substantially higher rate.

Related to treatment for OUD, we found that individuals with Medicaid were more likely to initiate MOUD at diagnosis compared with those with commercial insurance, although rates of treatment were low. Initiating MOUD had no effect on insurance transitions for those with commercial insurance but was associated with a 5.5% reduction in the probability of insurance transition for those receiving Medicaid. This may partly reflect differences between insurers in cost-sharing practices that could affect treatment retention,^20^ although findings on this topic are mixed.^31^

Our sensitivity analyses revealed several noteworthy findings. First, when altering our definition for insurance transition to allow for a 1-month lapse in insurance coverage, we found that the rates of insurance transitions decreased considerably for individuals covered by Medicaid but not by commercial insurance. We also found that nearly 50% of individuals with Medicaid who entered the uninsured/missing category reenrolled in Medicaid during the 12-month follow-up period (compared with only 20% of individuals with commercial insurance). This is consistent with known lapses in Medicaid enrollment due to annual eligibility redeterminations.^32^ Whether these brief lapses or transitions in insurance interrupt addiction treatment is a question that merits further inquiry.

Second, when we included more contemporary data, the insurance transition rates appeared higher for patients with commercial insurance than for Medicaid. The increase in insurance transitions for those with commercial insurance may be due to the Employee Retirement Income Security Act–related changes in reporting requirements to the APCD.^24^ The rate of insurance transitions in Medicaid was lower using more contemporary data (28.3% vs 30.7%), indicating that lapses in Medicaid insurance may have become less common over time.

Third, compared with a propensity score–matched cohort of patients with T2D, individuals with OUD had higher rates of insurance transitions, particularly for those with commercial insurance. The discrepancy in insurance transitions between individuals with OUD and T2D covered by commercial insurance may be associated with changes in employment associated with an OUD diagnosis. While changes in employment and insurance instability have been observed in other chronic conditions such as cancer,^33,34,35^ employer-based insurance may be particularly difficult to maintain for individuals with OUD. Active OUD may interfere with the ability to perform job functions, and individuals may also face stigma from employers due to drug use and treatment with MOUD.^36,37,38^

### Limitations

This study has several limitations. First, our analysis focused on insurance categories rather than individual insurance plans. As such, our insurance transition rates may be underestimates, as we would not detect individuals switching between plans within the same category of insurance^25^; in addition, the uninsured/missing category represents a heterogenous group, and the data limited our ability to distinguish insurance loss from other sources of transition, such as moving out of state. Second, we lacked data on important insurance characteristics that likely affect the treatment implications of insurance switching, such as network breadth and medication benefits. Third, our data are from a single state that expanded Medicaid eligibility and has high rates of insurance coverage, which may not generalize to other states.^39,40^ Fourth, while we can track longitudinal transitions in insurance, we do not assess whether these transitions affect treatment episodes, including disrupting or expanding access to MOUD. Fifth, we used a 180-day lookback period to define incident OUD, which may have captured some prevalent cases for individuals with limited contact with the medical system.

## Conclusions

In this longitudinal analysis of patients with newly diagnosed OUD, high rates of insurance transitions in the year after diagnosis, with differences by insurance type and by age and race and ethnicity were found. Given that insurance transitions may result in changes or loss of insurance coverage for highly efficacious medications for OUD, tracking changes in treatment outcomes related to insurance instability in this population is needed.

## Notes

Supplement 1. **eTable 1.** List of Data Sources From the Massachusetts Public Health Data Warehouse That Were Utilized in the Study **eTable 2.** List of ICD-9 and ICD-10 Codes Used to Identify New Diagnoses of Opioid Use Disorder and Type 2 Diabetes for Sensitivity Analyses **eTable 3.** Cohort Selection **eFigure 1.** Predicted Probability of Insurance Transition in the 12 Months After Opioid Use Disorder Diagnosis by a) Insurance Type, b) Age Category, c) Race/Ethnicity, and d) Whether an Individual Was Started on Medication for Opioid Use Disorder, 2014-2015 **eTable 4.** Types of Insurance Transitions Experienced in 12 Months Following Opioid Use Disorder Diagnosis for Individuals With Commercial or Medicaid Insurance, Using a 2-Month Gap in Coverage to Define Insurance Transition, 2014-2015 **eFigure 2.** Cumulative Incidence of Insurance Transitions in 12 Months After Opioid Use Disorder Diagnosis, by Commercial and Medicaid Insurance, Using a 2-Month Gap in Coverage to Define Insurance Transition, 2014-2015 **eTable 5.** Insurance Transitions Experienced After Transitioning to the Missing Category From Commercial vs Medicaid Insurance, 2014-2015 **eTable 6.** Patient Characteristics at the Time of Opioid Use Disorder Diagnosis, by Commercial vs Medicaid Insurance, Expanded Cohort From 2014-2020 **eTable 7.** Types of Insurance Transitions Experienced in 12 Months Following Opioid Use Disorder Diagnosis for Individuals With Commercial or Medicaid Insurance, Expanded Cohort From 2014-2020 **eTable 8.** Predicted Probability of Insurance Transition in the 12 Months After Opioid Use Disorder Diagnosis by Insurance Type and Select Patient Characteristics, Expanded Cohort From 2014-2020 **eTable 9.** Characteristics of the Propensity Score-Matched Study Cohorts at the Time Of Opioid Use Disorder or Type 2 Diabetes Diagnosis, by Commercial vs Medicaid Insurance, 2014-2015 **eTable 10.** Types of Insurance Transitions Experienced in 12 Months Following Opioid Use Disorder or Type 2 Diabetes Diagnosis for Individuals With Commercial or Medicaid Insurance, 2014-2015 **eFigure 3.** Cumulative Crude Incidence of Insurance Transitions in 12 Months After Opioid Use Disorder or Type 2 Diabetes Diagnosis, by Commercial and Medicaid Insurance, 2014-2015 **eTable 11.** Predicted Probability of Insurance Transition in the 12 Months After Opioid Use Disorder or Type 2 Diabetes Diagnosis by Insurance Type and Select Patient Characteristics, 2014-2015

Supplement 2.
Data Sharing Statement
