# Supplementary material for: Insurance Instability for Patients With Opioid Use Disorder in the Year After Diagnosis
Source: JAMA Health Forum. 2024 Jul 26;5(7):e242014. doi: 10.1001/jamahealthforum.2024.2014 (PMC11282441; doi:10.1001/jamahealthforum.2024.2014)
Supplement: Supplement 1. — eTable 1. List of Data Sources From the Massachusetts Public Health Data Warehouse That Were Utilized in the Study eTable 2. List of ICD-9 and ICD-10 Codes Used to Identify New Diagnoses of Opioid Use Disorder and Type 2 Diabetes for Sensitivity Analyses eTable 3. Cohort Selection eFigure 1. Predicted Probability of Insurance Transition in the 12 Months After Opioid Use Disorder Diagnosis by a) Insurance Type, b) Age Category, c) Race/Ethnicity, and d) Whether an Individual Was Started on Medication for Opioid Use Disorder, 2014-2015 eTable 4. Types of Insurance Transitions Experienced in 12 Months Following Opioid Use Disorder Diagnosis for Individuals With Commercial or Medicaid Insurance, Using a 2-Month Gap in Coverage to Define Insurance Transition, 2014-2015 eFigure 2. Cumulative Incidence of Insurance Transitions in 12 Months After Opioid Use Disorder Diagnosis, by Commercial and Medicaid Insurance, Using a 2-Month Gap in Coverage to Define Insurance Transition, 2014-2015 eTable 5. Insurance Transitions Experienced After Transitioning to the Missing Category From Commercial vs Medicaid Insurance, 2014-2015 eTable 6. Patient Characteristics at the Time of Opioid Use Disorder Diagnosis, by Commercial vs Medicaid Insurance, Expanded Cohort From 2014-2020 eTable 7. Types of Insurance Transitions Experienced in 12 Months Following Opioid Use Disorder Diagnosis for Individuals With Commercial or Medicaid Insurance, Expanded Cohort From 2014-2020 eTable 8. Predicted Probability of Insurance Transition in the 12 Months After Opioid Use Disorder Diagnosis by Insurance Type and Select Patient Characteristics, Expanded Cohort From 2014-2020 eTable 9. Characteristics of the Propensity Score-Matched Study Cohorts at the Time Of Opioid Use Disorder or Type 2 Diabetes Diagnosis, by Commercial vs Medicaid Insurance, 2014-2015 eTable 10. Types of Insurance Transitions Experienced in 12 Months Following Opioid Use Disorder or Type 2 Diabetes Diagnosis for Individuals With Comm [file jamahealthforum-e242014-s001.pdf]

Supplemental Online Content

Christine PJ, Goldman AL, Morgan JR, et al. Insurance instability for patients with opioid use disorder in the year after diagnosis. *JAMA Health Forum.* Published online July 26, 2024. doi:10.1001/jamahealthforum.2024.2014

eTable 1. List of Data Sources From the Massachusetts Public Health Data Warehouse That Were Utilized in the Study

eTable 2. List of ICD-9 and ICD-10 Codes Used to Identify New Diagnoses of Opioid Use Disorder and Type 2 Diabetes for Sensitivity Analyses

eTable 3. Cohort Selection

eFigure 1. Predicted Probability of Insurance Transition in the 12 Months After Opioid Use Disorder Diagnosis by a) Insurance Type, b) Age Category, c) Race/Ethnicity, and d) Whether an Individual Was Started on Medication for Opioid Use Disorder, 2014-2015

eTable 4. Types of Insurance Transitions Experienced in 12 Months Following Opioid Use Disorder Diagnosis for Individuals With Commercial or Medicaid

© 2024 Christine PJ et al. *JAMA Health Forum.*

Insurance, Using a 2-Month Gap in Coverage to Define Insurance Transition, 2014-2015

eFigure 2. Cumulative Incidence of Insurance Transitions in 12 Months After Opioid Use Disorder Diagnosis, by Commercial and Medicaid Insurance, Using a 2-Month Gap in Coverage to Define Insurance Transition, 2014-2015

eTable 5. Insurance Transitions Experienced After Transitioning to the Missing Category From Commercial vs Medicaid Insurance, 2014-2015

eTable 6. Patient Characteristics at the Time of Opioid Use Disorder Diagnosis, by Commercial vs Medicaid Insurance, Expanded Cohort From 2014-2020

eTable 7. Types of Insurance Transitions Experienced in 12 Months Following Opioid Use Disorder Diagnosis for Individuals With Commercial or Medicaid Insurance, Expanded Cohort From 2014-2020

eTable 8. Predicted Probability of Insurance Transition in the 12 Months After Opioid Use Disorder Diagnosis by Insurance Type and Select Patient Characteristics, Expanded Cohort From 2014-2020

eTable 9. Characteristics of the Propensity Score-Matched Study Cohorts at the Time Of Opioid Use Disorder or Type 2 Diabetes Diagnosis, by Commercial vs Medicaid Insurance, 2014-2015

eTable 10. Types of Insurance Transitions Experienced in 12 Months Following Opioid Use Disorder or Type 2 Diabetes Diagnosis for Individuals With Commercial or Medicaid Insurance, 2014-2015

eFigure 3. Cumulative Crude Incidence of Insurance Transitions in 12 Months After Opioid Use Disorder or Type 2 Diabetes Diagnosis, by Commercial and Medicaid Insurance, 2014-2015

eTable 11. Predicted Probability of Insurance Transition in the 12 Months After Opioid Use Disorder or Type 2 Diabetes Diagnosis by Insurance Type and Select Patient Characteristics, 2014-2015

This supplemental material has been provided by the authors to give readers additional information about their work.

© 2024 Christine PJ et al. *JAMA Health Forum.*

eTable 1: List of data sources from the Massachusetts Public Health Data Warehouse that were utilized in the study

Dataset name[a]	Dataset Abbreviation	Description
All-Payer Claims Data (APCD) – Medical[b]	PHDAPCD.MEDICAL	Identify OUD diagnoses<br>Identify methadone use within 30 days of OUD diagnosis
Case mix Emergency Department Visits[c]	PHDCM.ED&ED_DIAG	Identify OUD diagnoses
Case mix Inpatient Hospital Discharge[c]	PHDCM.HD&HD_DIAG	
Case mix Outpatient Observation Visit[c]	PHDCM.OO	
APCD – Member Eligibility Member Months	PHDAPCD.ME_MTH	Identify insurance status
APCD - Pharmacy	PHDAPCD.PHARMACY	Identify buprenorphine and naltrexone use within 30 day of OUD diagnosis
Prescription Monitoring Program	PHDPMP.PMP	Identify buprenorphine within 30 day of OUD diagnosis
Bureau of Substance Addiction Services	PHDBSAS.BSAS	Identify methadone use within 30 days of OUD diagnosis
Birth Records Infant/Child: Registry of Vital Records and Statistics	PHDBIRTH.BIRTH_INFANT	Identify pregnant status and birth
Birth Records Mom: Registry of Vital Records and Statistics	PHDBIRTH.BIRTH_MOM	
Registry of Vital Records and Statistics Mortality File	PHDDEATH.DEATH	Identify deaths for those transitioning to missing insurance category
Department of Correction	PHDDOC.DOC	Identify incarceration at baseline (180 days prior to index date), incarceration for those transit to missing insurance category
Houses of Correction	PHDHOC.HOC	
Spine Demographic Variables	PHDSPINE.DEMO	Identify race/ethnicity, sex and MA resident status
Opioid-related Overdoses and Overdose Deaths	PHDSPINE.OVERDOSE	Identify overdose at baseline (up to 180 days prior to index)

Abbreviations: APCD = All-Payer Claims Data; OUD = opioid use disorder; MA = Massachusetts
[a] For full details on datasets including brief descriptions, see https://www.mass.gov/doc/phd-datasets-brief-descriptions-pdf/download
[b] APCD claims include all insurance claims submitted by commercial insurers and public programs (Medicaid/MassHealth).
[c] Case mix data contains patient demographics, clinical characteristics, services provided, charges, and hospitals and practitioner
information. It includes data for all individuals receiving hospital care (ED, inpatient, or outpatient observation).

© 2024 Christine PJ et al. *JAMA Health Forum.*

eTable 2: List of ICD-9 and ICD-10 codes used to identify new diagnoses of opioid use disorder and type 2 diabetes for sensitivity analyses[a]

Condition	ICD9 Codes (starts with)	ICD10 Codes (starts with)
Opioid use disorder	3040	F111
	3047	F112
	3055	F119
Type 2 diabetes	250 + fifth digit 0 or 2	E11

[a] For a full list of codes to define the use of medications for opioid use disorder, including buprenorphine, methadone, and naltrexone, see the following link: https://osf.io/a7cz3/?view_only=f0f211ae6e7f428d880375f7d7b46a5d

© 2024 Christine PJ et al. *JAMA Health Forum.*

eTable 3: Cohort selection

Inclusion/Exclusion Criteria	Episodes Remaining	Individuals Remaining
Identify cases of OUD from Case Mix and APCD files	47001377	
Exclude those on long term opioid therapy	45857816	
Exclude those who had claims consistent with OUD in the prior 180 days (180-day washout period)	484716	
Limit timeframe for diagnosis from July 2014 to February 2019, and exclude those diagnosed in 2015	182634	
Keep only the first qualifying OUD diagnosis episode for each individual		137112
Exclude those <18 or >63 years old		127774
Exclude non-Massachusetts residents (sensitivity analysis with expanded cohort)		98979
Exclude those diagnosed after December 31, 2024 (primary analysis cohort)		20768

Abbreviations: OUD = opioid use disorder; APCD = All-Payer Claims Database

© 2024 Christine PJ et al. *JAMA Health Forum.*

eFigure 1: Predicted probability of insurance transition in the 12 months after opioid use disorder diagnosis by a) insurance type, b) age category, c) race/ethnicity, and d) whether an individual was started on medication for opioid use disorder, 2014-2015[e]

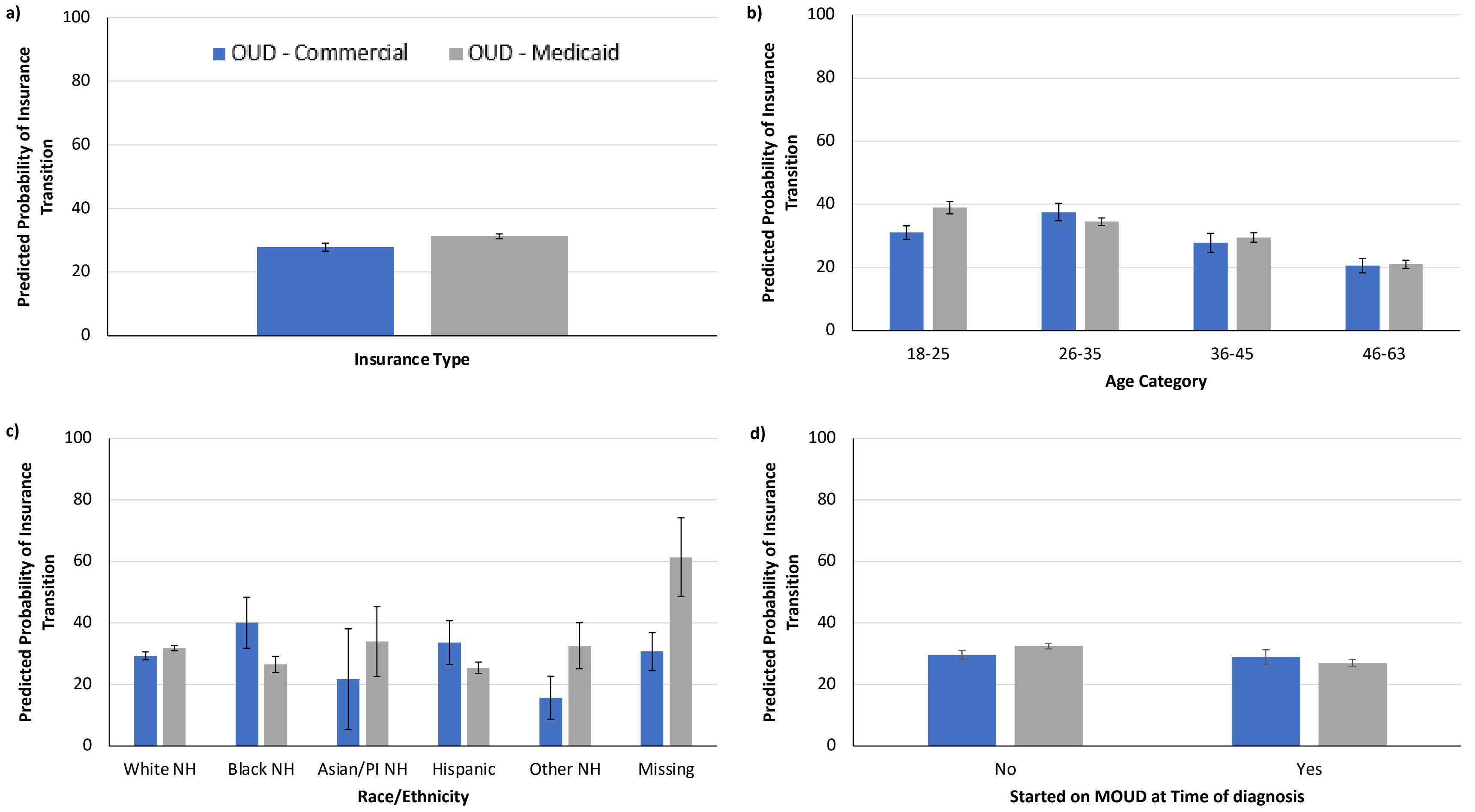

Abbreviations: OUD = opioid use disorder; NH = non-Hispanic; MOUD = medication for opioid use disorder
[e] Predicted probabilities generated from logistic regression models with insurance transition (yes/no) as the outcome. All models included age categories, race/ethnicity, pregnancy status, insurance type, and whether an individual started medication for opioid use disorder. Apart from figure a) which included individuals with both commercial and Medicaid insurance in the same model, all regression models were run separately by insurance type (commercial vs Medicaid).

© 2024 Christine PJ et al. *JAMA Health Forum.*

eTable 4: Types of insurance transitions experienced in 12 months following opioid use disorder diagnosis for individuals with commercial or Medicaid insurance, using a 2-month gap in coverage to define insurance transition, 2014-2015[a]

Insurance Transition, n (%)	Commercial at time of diagnosis	Medicaid at time of diagnosis
No transition	3713 (72.6)	11973 (76.5)
Any transition	1402 (27.4)	3680 (23.5)
Medicaid	566 (11.1)	NA
Commercial	NA	386 (2.5)
Medicare Advantage[b]	12 (0.2)	43 (0.3)
Other	168 (3.3)	617 (3.9)
Missing	656 (12.8)	2634 (16.8)
Died[c]	12 (1.8)	104 (3.9)
Incarcerated[c]	*	182 (6.9)

Abbreviations: NA = not applicable; * = suppressed data (0 > n < 11)

[a] Insurance transition defined as a change from baseline insurance type (either Commercial or Medicaid) to either a) a different insurance type, or b) "missing" insurance, which could occur due to moving away from Massachusetts, becoming uninsured, dying, or becoming incarcerated. The "other" category includes emergency Medicaid, Veterans Affairs, and worker's compensation. In main analyses, insurance transition corresponded to any 1-month transition. In this table, patients are allowed to move to the "missing" category for up to 1 month and return to their original insurance the following month without counting as a transition.

[b] Study population limited to individuals less than 64 years of age at time of diagnosis. Insurance transitions to Medicare therefore likely indicate enrollment due to the presence of a qualifying disability, end stage renal disease, or amyotrophic lateral sclerosis.

[c] Denominator for "Died" and "Incarcerated" is the number of missing rather than the number with a particular insurance.

© 2024 Christine PJ et al. *JAMA Health Forum.*

eFigure 2: Cumulative incidence of insurance transitions in 12 months after opioid use disorder diagnosis, by commercial and Medicaid insurance, using a 2-month gap in coverage to define insurance transition, 2014-2015[a]

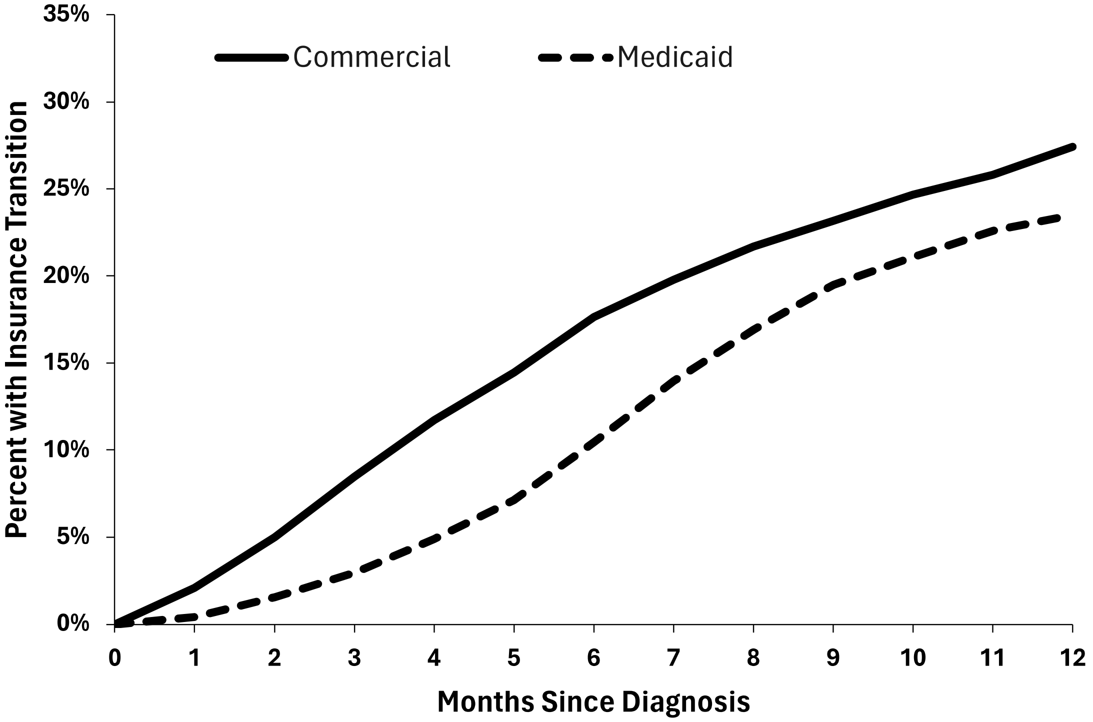

[a] Insurance transition defined as a change from baseline insurance type (either Commercial or Medicaid) to either a) a different type of insurance, or b) “missing” insurance, which could occur due to moving away from Massachusetts, becoming uninsured, dying, or becoming incarcerated. In main analyses, insurance transition corresponded to any 1-month transition. In this figure, patients are allowed to move to the “missing” category for up to 1 month and return to their original insurance the following month without counting as a transition.

© 2024 Christine PJ et al. *JAMA Health Forum.*

eTable 5: Insurance transitions experienced after transitioning to the missing category from commercial vs Medicaid insurance, 2014-2015[a]

Insurance Transition After Entering Missing Category, n (%)	Commercial at time of diagnosis	Medicaid at time of diagnosis
Stayed missing	638 (74.6)	1822 (47.7)
Medicaid	39 (4.6)	1896 (49.6)
Commercial	175 (20.5)	93 (2.4)
Medicare Advantage	0 (0)	0 (0)
Other	*	12 (0.3)

Abbreviations: NA = not applicable; * = suppressed data (0 > n < 11)

[a] Table includes individuals initially diagnosed with OUD and covered by commercial or Medicaid insurance who entered the "missing" category at any time during the 12-month follow up. "Missing" insurance could occur due to moving away from Massachusetts, becoming uninsured, dying, or becoming incarcerated. The "other" category includes emergency Medicaid, Veterans Affairs, and worker's compensation. Frequencies represent whether an individual who entered the missing category subsequently transitioned to a different category sometime during the 12-month follow up period.

© 2024 Christine PJ et al. *JAMA Health Forum.*

eTable 6: Patient characteristics at the time of opioid use disorder diagnosis, by commercial vs Medicaid insurance, expanded cohort from 2014-2020[a]

Characteristic	Commercial	Medicaid
Sample size, n (%)	25206 (25.5)	73773 (74.5)
Age category, n (%)		
18-25	5182 (20.6)	8406 (11.4)
26-35	7461 (29.6)	26352 (35.7)
36-45	5261 (20.9)	17047 (23.1)
46-63[b]	7302 (29.0)	21968 (29.8)
Female, n (%)	8639 (34.3)	30016 (40.7)
Race/ethnicity, n (%)		
White non-Hispanic	20148 (79.9)	55877 (75.7)
Black non-Hispanic	652 (2.6)	6077 (8.2)
Asia/Pacific Islander non-Hispanic	113 (0.5)	320 (0.4)
Hispanic	1000 (4.0)	10342 (14.0)
Other non-Hispanic	229 (0.9)	623 (0.8)
Missing	3064 (12.2)	534 (0.7)
Pregnant, n (%)	18 (0.1)	144 (0.2)
Number of hospitalizations in last 180 days, n (%)		
0	23169 (91.9)	63274 (85.8)
1	1411 (5.6)	6597 (8.9)
2	358 (1.4)	1942 (2.6)
3+	268 (1.1)	1960 (2.7)
Number of emergency department visits in last 180 days, n (%)		
0	19760 (78.4)	41389 (56.1)
1	3577 (14.2)	16242 (22.0)
2	1059 (4.2)	7376 (10.0)
3+	810 (3.2)	8766 (11.9)
Incarcerated in last 180 days, n (%)[c]	384 (1.5)	5281 (7.2)
Opioid overdose in last 180 days, n (%)	421 (1.7)	2381 (3.2)
Started medication for OUD at time of diagnosis, n (%)		
None	15493 (61.5)	45762 (62.0)
Methadone	1529 (6.1)	7110 (9.6)
Buprenorphine	6650 (26.4)	16831 (22.8)
Naltrexone	1534 (6.1)	4070 (5.5)

Abbreviations: OUD = opioid use disorder

[a] Cohort excludes individuals diagnosed in 2015 due to the *Gobeille v Liberty Mutual Insurance Co* Supreme Court ruling which specified that self-employed employer plans were no longer required to report claims data to state All-Payer Claims Databases.

[b] Cohort limited to adults 18-63 years old. Upper age limit of 63 was imposed to avoid counting insurance transitions to Medicare at age 65.

[c] Incarceration data includes all state prisons and most county jails (10 out of 13), except for Bristol, Barnstable/Nantucket, and Duke counties.

© 2024 Christine PJ et al. *JAMA Health Forum.*

eTable 7: Types of insurance transitions experienced in 12 months following opioid use disorder diagnosis for individuals with commercial or Medicaid insurance, expanded cohort from 2014-2020[a]

Insurance Transition, n (%)	Commercial at time of diagnosis	Medicaid at time of diagnosis
No transition	16640 (66.0)	52910 (71.7)
Any transition	8566 (34.0)	20863 (28.3)
Medicaid	1699 (6.7)	NA
Commercial	NA	1387 (1.9)
Medicare Advantage[b]	43 (0.2)	273 (0.4)
Other	884 (3.5)	2197 (3.0)
Missing	5940 (23.6)	17006 (23.1)
Died[c]	45 (0.8)	529 (3.1)
Incarcerated[c]	36 (0.6)	427 (2.5)

Abbreviations: NA = not applicable; * = suppressed data (0 > n < 11)

[a] Cohort excludes individuals diagnosed in 2015 due to the *Gobeille v Liberty Mutual Insurance Co* Supreme Court ruling which specified that commercial insurers were no longer required to report healthcare claims data to state All-Payer Claims Databases. Insurance transition defined as a change from baseline insurance type (either Commercial or Medicaid) to either a) a different insurance type, or b) "missing" insurance, which could occur due to moving away from Massachusetts, becoming uninsured, dying, becoming incarcerated, or switching to a commercial insurance plan that does not report to the Massachusetts All-Payer Claims Database. The "other" category includes emergency Medicaid, Veterans Affairs, and worker's compensation.

[b] Study population limited to individuals less than 64 years of age at time of diagnosis. Insurance transitions to Medicare therefore likely indicate enrollment due to the presence of a qualifying disability, end stage renal disease, or amyotrophic lateral sclerosis.

[c] Denominator for "Died" and "Incarcerated" is the number of missing rather than the number with a particular insurance. Incarceration data includes all state prisons and most county jails (10 out of 13), except for Bristol, Barnstable/Nantucket, and Duke counties.

© 2024 Christine PJ et al. *JAMA Health Forum.*

eTable 8: Predicted probability of insurance transition in the 12 months after opioid use disorder diagnosis by insurance type and select patient characteristics, expanded cohort from 2014-2020[a]

	Probability of Insurance Transition (95% CI)	
**Characteristic**	**Commercial at time of diagnosis**	**Medicaid at time of diagnosis**
Overall	32.9 (32.3 to 33.5)	28.6 (28.3 to 29.0)
Age category		
18-25	33.5 (32.2 to 34.8)	34.5 (33.5 to 35.5)
26-35	42.3 (41.2 to 43.4)	32.2 (31.6 to 32.7)
36-45	33.5 (32.2 to 34.8)	27.7 (27.0 to 28.3)
46-63	26.2 (25.1 to 27.2)	21.7 (21.1 to 22.2)
Race/ethnicity		
White non-Hispanic	33.0 (32.3 to 33.6)	29.1 (28.7 to 29.5)
Black non-Hispanic	42.5 (38.8 to 46.3)	25.1 (24.0 to 26.2)
Asian/Pacific Islander non-Hispanic	35.4 (26.7 to 44.1)	31.4 (26.5 to 36.4)
Hispanic	39.4 (36.3 to 42.4)	24.5 (23.7 to 25.4)
Other non-Hispanic	37.1 (31.0 to 43.2)	29.8 (26.3 to 33.2)
Missing	36.6 (34.9 to 38.3)	47.5 (43.4 to 51.5)
Started medication for OUD at time of diagnosis		
No	33.9 (33.1 to 34.6)	29.8 (29.4 to 30.2)
Yes	34.1 (33.2 to 35.1)	25.8 (25.3 to 26.3)

Abbreviations: OUD = opioid use disorder; CI = confidence interval; NA = non-applicable

[a] Predicted probabilities generated from logistic regression models with insurance transition (yes/no) as the outcome. All models included age categories, race/ethnicity, pregnancy status, and insurance type, and whether an individual started medication for OUD at the time of diagnosis. Apart from generating the overall probabilities for insurance transitions, which included individuals with both commercial and Medicaid insurance in the same model, all regression models were run separately by insurance type (commercial vs Medicaid).

© 2024 Christine PJ et al. *JAMA Health Forum.*

eTable 9: Characteristics of the propensity score-matched study cohorts at the time of opioid use disorder or type 2 diabetes diagnosis, by commercial vs Medicaid insurance, 2014-2015[a]

	Opioid Use Disorder		Type 2 Diabetes	
**Characteristic**	**Commercial**	**Medicaid**	**Commercial**	**Medicaid**
Sample size, n (%)	4213	9602	4213	9602
Age category, n (%)				
18-25	1094 (26.0)	836 (8.7)	830 (19.7)	761 (7.9)
26-35	1046 (24.8)	2691 (28.0)	1264 (30.0)	2072 (21.6)
36-45	832 (19.8)	2746 (28.6)	819 (19.4)	2531 (26.4)
46-63[b]	1241 (29.5)	3329 (34.7)	1300 (30.9)	4238 (44.1)
Female, n (%)	1505 (35.7)	4124 (43.0)	1774 (42.1)	4096 (42.7)
Race/ethnicity, n (%)				
White non-Hispanic	3604 (85.5)	6676 (69.5)	3529 (83.8)	6384 (66.5)
Black non-Hispanic	128 (3.0)	991 (10.3)	139 (3.3)	1133 (11.8)
Asia/Pacific Islander non-Hispanic	23 (0.6)	61 (0.6)	24 (0.6)	45 (0.5)
Hispanic	138 (3.3)	1700 (17.7)	157 (3.7)	1878 (19.6)
Other non-Hispanic	106 (2.5)	120 (1.3)	96 (2.3)	98 (1.0)
Missing	214 (5.1)	54 (0.6)	268 (6.4)	64 (0.7)
Pregnant, n (%)	*	24 (0.3)	11 (0.3)	24 (0.3)
Number of hospitalizations in last 180 days, n (%)				
0	3874 (92.0)	8256 (86.0)	4033 (95.7)	8681 (90.4)
1	224 (5.3)	840 (8.8)	137 (3.3)	570 (5.9)
2	61 (1.5)	261 (2.7)	26 (0.6)	183 (1.9)
3+	54 (1.3)	245 (2.6)	17 (0.4)	168 (1.8)
Number of emergency department visits in last 180 days, n (%)				
0	3336 (79.2)	5509 (57.4)	3754 (89.1)	7042 (73.3)
1	569 (13.5)	2021 (21.1)	351 (8.3)	1480 (15.4)
2	166 (3.9)	955 (10.0)	63 (1.5)	507 (5.3)
3+	142 (3.4)	1117 (11.6)	45 (1.1)	573 (6.0)
Incarcerated in last 180 days, n (%)[c]	18 (0.4)	409 (4.3)	12 (0.3)	178 (1.9)
Opioid overdose in last 180 days, n (%)	44 (1.0)	194 (2.0)	NA	NA
Started medication for OUD at time of diagnosis, n (%)				
None	2981 (70.8)	6371 (66.4)	NA	NA
Methadone	96 (2.3)	850 (8.9)	NA	NA
Buprenorphine	962 (22.8)	2098 (21.9)	NA	NA
Naltrexone	178 (4.2)	305 (3.2)	NA	NA

Abbreviations: OUD = opioid use disorder; * = suppressed data (0 > n < 11)

[a] Cohort limited to adults 18-63 years old. Upper age limit of 63 was imposed to avoid counting insurance transitions to Medicare at age 65. Propensity scores were created using logistic regression to model the probability of having OUD or type 2 diabetes. Propensity score models included age, sex, race/ethnicity, disability status (yes/no), highest education level, homelessness (yes/no), and history of incarceration (yes/no). Matching was completed using nearest-neighbor matching with a caliper of ± 0.05 (± 5% probability). Propensity score models were run separately for Medicaid and commercial insurance.

© 2024 Christine PJ et al. *JAMA Health Forum.*

[b] Incarceration data includes all state prisons and most county jails (10 out of 13), except for Bristol, Barnstable/Nantucket, and Duke counties.

© 2024 Christine PJ et al. *JAMA Health Forum.*

eTable 10: Types of insurance transitions experienced in 12 months following opioid use disorder or type 2 diabetes diagnosis for individuals with commercial or Medicaid insurance, 2014-2015[a]

	Opioid Use Disorder		Type 2 Diabetes	
**Insurance Transition, n (%)**	**Commercial at time of diagnosis**	**Medicaid at time of diagnosis**	**Commercial at time of diagnosis**	**Medicaid at time of diagnosis**
No transition	3084 (73.2)	6883 (71.7)	3476 (82.5)	7171 (74.7)
Any transition	1129 (26.8)	2719 (28.3)	737 (17.5)	2431 (25.3)
Medicaid	307 (7.3)	NA	187 (4.4)	NA
Commercial	NA	242 (2.5)	NA	426 (4.4)
Medicare Advantage[b]	*	35 (0.4)	*	36 (0.4)
Other	135 (3.2)	384 (4.0)	106 (2.5)	609 (6.3)
Missing	677 (16.1)	2058 (21.4)	437 (10.4)	1360 (14.2)
Died[c]	13 (1.9)	86 (4.2)	*	64 (4.7)
Incarcerated[c]	*	36 (1.7)	*	13 (1.0)

Abbreviations: NA = not applicable; * = suppressed data (0 > n < 11)

[a] Insurance transition defined as a change from baseline insurance type (either Commercial or Medicaid) to either a) a different insurance type, or b) "missing" insurance, which could occur due to moving away from Massachusetts, becoming uninsured, dying, or becoming incarcerated. The "other" category includes emergency Medicaid, Veterans Affairs, and worker's compensation.

[b] Study population limited to individuals less than 64 years of age at time of diagnosis. Insurance transitions to Medicare therefore likely indicate enrollment due to the presence of a qualifying disability, end stage renal disease, or amyotrophic lateral sclerosis. Cohorts of opioid use disorder and type 2 diabetes patients are propensity score-matched separately by insurance type.

[c] Denominator for "Died" and "Incarcerated" is the number of missing rather than the number with a particular insurance. Incarceration data includes all state prisons and most county jails (10 out of 13), except for Bristol, Barnstable/Nantucket, and Duke counties.

© 2024 Christine PJ et al. *JAMA Health Forum.*

eFigure 3: Cumulative crude incidence of insurance transitions in 12 months after opioid use disorder or type 2 diabetes diagnosis, by commercial and Medicaid insurance, 2014-2015[a]

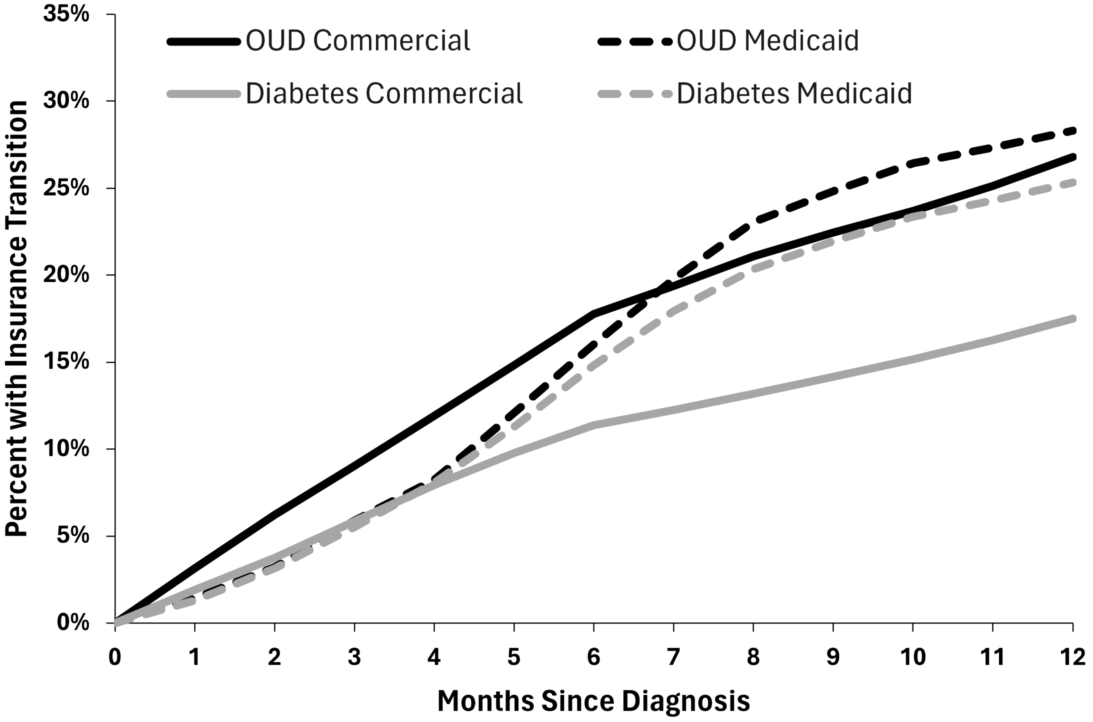

[a] Cohorts of opioid use disorder and type 2 diabetes patients are propensity score-matched separately by insurance type. Insurance transition defined as a change from baseline insurance type (either Commercial or Medicaid) to either a) a different type of insurance, or b) "missing" insurance, which could occur due to moving away from Massachusetts, becoming uninsured, dying, or becoming incarcerated. In main analyses, insurance transition corresponded to any 1-month transition. In this figure, patients are allowed to move to the "missing" category for up to 1 month and return to their original insurance the following month without counting as a transition.

© 2024 Christine PJ et al. *JAMA Health Forum.*

eTable 11: Predicted probability of insurance transition in the 12 months after opioid use disorder or type 2 diabetes diagnosis by insurance type and select patient characteristics, 2014-2015[a]

	Probability of Insurance Transition (95% CI)			
	Opioid Use Disorder		Type 2 Diabetes	
**Characteristic**	**Commercial at time of diagnosis**	**Medicaid at time of diagnosis**	**Commercial at time of diagnosis**	**Medicaid at time of diagnosis**
Overall	24.5 (23.2 to 25.9)	29.4 (28.5 to 30.4)	15.7 (14.6 to 16.8)	26.4 (25.5 to 27.4)
Age category				
18-25	27.3 (24.7 to 30.0)	40.5 (37.2 to 43.8)	20.4 (17.7 to 23.1)	29.1 (25.7 to 32.4)
26-35	34.2 (31.3 to 37.1)	35.0 (33.2 to 36.8)	19.8 (17.6 to 22.0)	29.4 (27.4 to 31.4)
36-45	26.9 (23.8 to 29.9)	28.1 (26.5 to 29.8)	17.8 (15.2 to 20.4)	25.6 (23.9 to 27.3)
46-63	20.2 (18.0 to 22.5)	20.2 (18.9 to 21.6)	13.1 (11.2 to 14.9)	22.8 (21.5 to 24.0)
Race/ethnicity				
White non-Hispanic	26.5 (25.1 to 27.9)	30.2 (29.1 to 31.3)	16.4 (15.2 to 17.6)	27.0 (25.9 to 28.1)
Black non-Hispanic	37.3 (28.9 to 45.7)	23.4 (20.9 to 26.0)	20.4 (13.7 to 27.1)	21.3 (18.9 to 23.6)
Asian/Pacific Islander non-Hispanic	18.0 (2.2 to 33.8)	30.5 (19.8 to 41.2)	37.1 (18.1 to 56.1)	14.8 (4.7 to 24.9)
Hispanic	30.3 (22.7 to 37.9)	22.9 (21.0 to 24.8)	22.5 (16.1 to 28.9)	20.7 (18.9 to 22.5)
Other non-Hispanic	15.2 (8.4 to 22.0)	30.6 (22.9 to 38.4)	15.3 (8.1 to 22.5)	31.6 (22.6 to 40.5)
Missing	30.2 (24.0 to 36.4)	58.1 (45.0 to 71.1)	26.3 (21.1 to 31.6)	62.1 (49.9 to 74.2)
Started medication for OUD at time of diagnosis				
No	27.4 (25.8 to 29.0)	30.3 (29.1 to 31.4)	NA	NA
Yes	25.3 (22.8 to 27.7)	24.7 (23.2 to 26.1)	NA	NA

Abbreviations: OUD = opioid use disorder; CI = confidence interval; NA = non-applicable
[a] Cohorts of opioid use disorder and type 2 diabetes patients are propensity score-matched separately by insurance type. Predicted probabilities generated from logistic regression models with insurance transition (yes/no) as the outcome. All models included age categories, race/ethnicity, pregnancy status, and insurance type, and for OUD, whether an individual started medication for OUD at the time of diagnosis. Apart from generating the overall probabilities for insurance transitions, which included individuals with both commercial and Medicaid insurance in the same model, all regression models were run separately by insurance type (commercial vs Medicaid).

© 2024 Christine PJ et al. *JAMA Health Forum.*
