# Supplementary material for: Insurance Instability for Patients With Opioid Use Disorder in the Year After Diagnosis
Source: JAMA Health Forum. 2024 Jul 26;5(7):e242014. doi: 10.1001/jamahealthforum.2024.2014 (PMC11282441; doi:10.1001/jamahealthforum.2024.2014)
Supplement: Supplement 2. — Data Sharing Statement [file jamahealthforum-e242014-s002.pdf]

Data Sharing Statement

Christine. Insurance Instability for Patients With Opioid Use Disorder in the Year After Diagnosis. *JAMA Health Forum*. Published July 26, 2024.
doi:10.1001/jamahealthforum.2024.2014

Data
Data available: No

Additional Information
Explanation for why data not available: The data from this study comes from the Massachusetts Public Health Data Warehouse, which is housed within the Massachusetts Department of Public Health. External researchers can apply to work with the data but the raw data is not publicly available to share as part of a manuscript.
